# A high triglyceride-glucose index is associated with left ventricular dysfunction and atherosclerosis

**DOI:** 10.7150/ijms.53920

**Published:** 2021-01-01

**Authors:** Tai-Hua Chiu, Hui-Ju Tsai, Hsin-Ying Clair Chiou, Pei-Yu Wu, Jiun-Chi Huang, Szu-Chia Chen

**Affiliations:** 1 Department of General Medicine, Kaohsiung Medical University Hospital, Kaohsiung, Taiwan; 2 Department of Family Medicine, Kaohsiung Municipal Ta-Tung Hospital, Kaohsiung Medical University Hospital, Kaohsiung Medical University, Kaohsiung, Taiwan; 3 Graduate Institute of Clinical Medicine, College of Medicine, Kaohsiung Medical University, Kaohsiung, Taiwan; 4 Research Center for Environmental Medicine, Kaohsiung Medical University, Kaohsiung, Taiwan; 5 Teaching and Research Center, Kaohsiung Municipal Siaogang Hospital, Kaohsiung Medical University, Kaohsiung, Taiwan; 6 Division of Nephrology, Department of Internal Medicine, Kaohsiung Medical University Hospital, Kaohsiung Medical University, Kaohsiung, Taiwan; 7 Department of Internal Medicine, Kaohsiung Municipal Siaogang Hospital, Kaohsiung Medical University, Kaohsiung, Taiwan; 8 Faculty of Medicine, College of Medicine, Kaohsiung Medical University, Kaohsiung, Taiwan

**Keywords:** triglyceride-glucose index, left ventricular dysfunction, left ventricular mass, atherosclerosis

## Abstract

**Background:** The triglyceride-glucose (TyG) index has been reported to be a simple and reliable surrogate marker of insulin resistance. The aim of this study was to investigate associations between the TyG index and echocardiographic parameters including left ventricular mass (LVM), left atrial diameter (LAD) and left ventricular ejection fraction (LVEF), and markers of peripheral artery disease, ankle-brachial index (ABI) and brachial-ankle pulse wave velocity (baPWV).

**Methods:** A total of 823 (483 males and 340 females) patients were enrolled from 2007 to 2011 at a regional hospital in southern Taiwan. Multivariable stepwise linear regression analysis was performed to identify the factors related to echocardiographic parameters and peripheral artery disease.

**Results:** The patients were stratified into four groups according to TyG index quartile. Multivariable stepwise linear regression analysis showed that a higher TyG index was associated with elevated observed/predicted LVM (*p* = 0.081), increased LAD (*p* = 0.004), decreased LVEF (*p* = 0.003) and lower ABI (*p* = 0.030), but not observed/predicted LVM and baPWV.

**Conclusions:** A high TyG index was significantly associated with high LAD, low LVEF and low ABI. However, the TyG index was not significantly associated with inappropriate LVM or baPWV. The results suggest that the TyG index, as a simple indicator of insulin resistance, may reflect cardiac remodeling and dysfunction and atherosclerosis.

## Introduction

Cardiovascular (CV) diseases account for about one-third of all deaths mortality globally, and they have become a major public health burden over the past several decades 1. Abnormal adiposity and derangement of glucose metabolism are two important components of metabolic syndrome, and they have been suggested to play major roles in the development of CV diseases 2,3. The triglyceride-glucose (TyG) index is derived from both fasting triglycerides and glucose, and it has been shown to be a novel marker of insulin resistance (IR) and to have better predictive value than traditional parameters 4,5. In addition, previous studies have reported associations between the TyG index and various CV abnormalities, including hypertension 6, arterial stiffness 7 and coronary artery calcification 8. Moreover, higher TyG index levels have also been associated with a poor prognosis in patients with acute ST-elevation myocardial infarction 9, higher CV mortality in patients starting peritoneal dialysis 10, and future adverse CV events in patients with diabetes mellitus (DM) and acute coronary syndrome 11. Our previous study revealed an association between the TyG index and an increased risk of CV events in patients with type 2 DM 12, and another study reported that a high TyG index was associated with micro- and macro-angiopathies in patients with type 2 DM 13. However, the exact mechanism underlying the association is still unclear.

Vascular screening devices have been developed to measure both ankle-brachial index (ABI) and brachial-ankle pulse wave velocity (baPWV), which have been shown to be good markers of atherosclerosis and arterial stiffness, respectively 14,15. A recent study of Chinese elderly by Zhao *et al.* investigated associations between the TyG index and parameters of vascular damage, including ABI, baPWV, carotid-femoral pulse wave velocity (cfPWV), carotid intima-media thickness and carotid plaque, and found a significant association between an elevated TyG index and a higher risk of nephric microvascular damage and arterial stiffness 16. However, few studies have explored associations between the TyG index and echocardiographic parameters, which can be used to evaluate cardiac function and possibly predict future CV risk 17,18. Thus the aim of this study was to investigate associations between the TyG index and echocardiographic parameters including left ventricular mass (LVM), left atrial diameter (LAD) and left ventricular ejection fraction (LVEF), and markers of peripheral artery disease, ABI and baPWV.

## Materials and methods

### Study Patients

The study design has been described previously 19. In brief, we enrolled patients who underwent echocardiographic examinations from 2007 to 2011 at a regional hospital in southern Taiwan. Patients with atrial fibrillation, significant ankle edema, significant aortic or mitral valve diseases and poor image quality were excluded. A total of 823 participants (mean age 61.3 ± 13.2 years, 483 males and 340 females) were included in this study. The protocol was approved by the Institutional Review Board of Kaohsiung Medical University Hospital, and all enrolled patients gave written informed consent.

### Evaluation of Cardiac Structure and Function

Echocardiographic examinations were performed by one experienced cardiologist using a VIVID 7 system (General Electric Medical Systems, Horten, Norway) with the participant placed in the left decubitus position breathing quietly. The cardiologist was blinded to the other data. Two-dimensional and 2-dimensionally guided standard M-mode images were obtained, and echocardiographic parameters including LAD, left ventricular posterior wall thickness in diastole (LVPWTd), left ventricular internal diameter in diastole (LVIDd), interventricular septal wall thickness in diastole (IVSTd), transmitral E wave velocity, transmitral A wave velocity, and E-wave deceleration time were measured. Left ventricular systolic function was assessed according to the LVEF. LVM was calculated using a modification of Devereux's method as follows: LVM = 1.04 x ([IVSTd + LVIDd + LVPWTd]^3^ - LVIDd^3^) - 13.6 g 20. Left ventricular mass index (LVMI) was calculated as LVM/body surface area. Left ventricular hypertrophy (LVH) was defined according to the 2007 European Society of Hypertension/European Society of Cardiology guidelines 21. Inappropriate LVM was defined as observed/predicted LVM. Predicted LVM was calculated as: predicted LVM = 55.37 + 6.64 x height (m^2.7^) + 0.64 x stroke work - 18.07 x sex (in which sex was coded as male = 1 and female = 2) 22. Stroke work in gram-meters was calculated as systolic blood pressure x stroke volume x 0.0144. 'Inappropriate' LVM was defined as observed/predicted LVM > 128% 22,23.

### Assessment of baPWV and ABI

For baPWV, pulse waves were recorded in both brachial and tibial arteries to calculate the transmission time (ΔTba), which was defined as the time between the initial increase in ankle and brachial waveforms 15,24,25. The distance from the ankle to brachium was defined as the transmission distance and calculated according to body height. The path length from the suprasternal notch to the brachium (Lb) was calculated as: Lb = 0.2195 x height of the patient (in cm) - 2.0734. The path length from the suprasternal notch to the ankle (La) was calculated as: La = 0.8129 x height of the patient (in cm) + 12.328. BaPWV was then calculated as: baPWV = (La - Lb)/ΔTba. The higher of bilateral baPWV values was used for analysis. ABI was measured using an ABI-form device, which measured blood pressures (BPs) in both arms and ankles using an oscillometric method, and calculated as: ankle systolic BP/arm systolic BP, with only the lower ankle systolic BP value being used. ABI was measured once in each patient.

### Collection of Demographic, Medical and Laboratory Data

Demographic and medical data including age, sex, and comorbid conditions were obtained from medical records or interviews with the patients. Body mass index (BMI) was calculated as weight/height squared (kg/m^2^). Laboratory data including fasting glucose, triglycerides, total cholesterol, creatinine, and hematocrit were measured from fasting blood samples using an autoanalyzer (D-68298 Mannheim COBAS Integra 400; Roche Diagnostics GmbH, Mannheim, Germany). Blood samples were obtained within 1 month of enrollment. Serum creatinine was measured using the compensated Jaffé method on the Integra 400 system (Roche Diagnostics GmbH) with calibration traceable to isotope-dilution mass spectrometry 26. Estimated glomerular filtration rate (eGFR) was calculated using the four-variable Modification of Diet in Renal Disease equation 27.

### Statistical Analysis

The patients were stratified into four groups according to TyG index quartile. Data are expressed as percentages for categorical variables or mean ± SD for continuous variables. Comparisons among groups were performed using one-way analysis of variance (ANOVA) followed by a post hoc test with Bonferroni correction. Multivariate stepwise linear regression analysis was used to identify the factors associated with echocardiographic parameters (observed/predicted LVM, LAD and LVEF) and peripheral artery disease (ABI and baPWV). Statistical analysis was performed using SPSS version 19.0 for Windows (IBM Corp, Armonk, NY). A difference was considered to be significant if the P value was < 0.05.

## Results

This study enrolled 823 participants stratified into four groups by TyG index quartile, with 207, 207, 206 and 203 in each group, respectively. Table 1 shows comparisons of clinical characteristics among the study groups. The cutoff values of the TyG index quartiles were < 8.4, ≥ 8.4 to < 8.8, ≥ 8.8 to < 9.3, and ≥ 9.3, respectively. The mean values ± standard deviations of the TyG index in the four groups were 8.1 ± 0.2, 8.6 ± 0.1, 9.0 ± 0.1, and 9.8 ± 0.5, respectively. Compared to the patients in quartile 1, those in quartile 4 were younger, and had a higher prevalence of DM, higher BMI, higher cholesterol, higher observed/predicted LVM and higher LAD.

### Determinants of Observed/Predicted LVM, LAD and LVEF in the Study Patients

Table 2 summarizes the possible determinants of observed/predicted LVM, LAD and LVEF in our study patients. After adjusting for age, sex, history of DM and hypertension, mean arterial pressure, hematocrit, eGFR and total cholesterol, multivariate stepwise linear regression analysis revealed that young age (*p* = 0.013), DM (*p* = 0.021), low mean arterial pressure (*p* < 0.001) and low eGFR (*p* < 0.001) were significantly associated with high observed/predicted LVM, but that TyG index was not (unstandardized coefficient β, 4.401; 95% CI, -0.540 to 9.342; *p* = 0.081). Regarding LAD, a high TyG index (unstandardized coefficient β, 4.401; 95% CI, -0.540 to 9.342; *p* = 0.081), male sex (*p* < 0.001), and low eGFR (*p* < 0.001) were significantly correlated with high LAD. In addition, a high TyG index (unstandardized coefficient β, -2.012; 95% CI, -3.338 to -0.686; *p* = 0.003), young age (*p* = 0.026), male (*p* = 0.005), no history of hypertension (*p* < 0.001), coronary artery disease (*p* < 0.001) and low eGFR (*p* < 0.001) were significantly correlated with low LVEF.

### Determinants of ABI and baPWV in the Study Patients

Table 3 presents the risk factors for peripheral artery disease. In multivariate stepwise linear regression analysis, a high TyG index (unstandardized coefficient β, -0.147; 95% CI, -0.279 to -0.014; *p* = 0.030), old age (*p* < 0.001), male sex (*p* = 0.022), coronary artery disease (*p* = 0.012) and high total cholesterol (*p* = 0.038) were significantly correlated with low ABI. Regarding baPWV, old age (*p* < 0.001), diabetes (*p* < 0.001) and high mean arterial pressure (*p* < 0.001) were significantly correlated with high baPWV, but TyG index was not.

## Discussion

In this study, we investigated associations between the TyG index and echocardiographic parameters of cardiac remodeling and dysfunction and indices of atherosclerosis and arterial stiffness. The results showed that a high TyG index was associated with high LAD, low LVEF and low ABI, while observed/predicted LVM and baPWV failed to show significant associations with TyG index.

The etiology of LVH includes both the impact of hemodynamic overload on the heart and other non-hemodynamic mechanisms, among which metabolic factors have been suggested to play a major role in the development of LVH 28. Increasing evidence has demonstrated an association between impaired glucose metabolism and LVM. In an* in vivo* study on insulin-treated rats, Samuelsson *et al.* noted myocyte hypertrophy and increased interstitial fibrosis in histochemical examinations 29. In addition, in a clinical study of 2623 subjects from the Framingham Study, Rutter *et al.* reported that increases in LVM and wall thickness were positively associated with worsening glucose intolerance 30. In addition, in a study of 820 elderly individuals, Sundström *et al.* reported that IR determined by the homeostasis model assessment formula (HOMA-IR) was related to LVM and relative wall thickness 31. In another longitudinal study of 627 participants, Cauwenberghs *et al.* found that hyperinsulinemia was associated with a greater increase in LVM 32. Inappropriate LVM is defined as an increase in LVM that exceeds the compensatory need of hemodynamics 33. In the present study, we found that a high TyG index was borderline significantly associated with high observed/predicted LVM. Considering that the TyG index is a recognized surrogate of IR 4, our findings are consistent with previous reports 30-32. Several mechanisms have been proposed to explain the relationship between IR and increase in LVM. High levels of insulin *per se* may interact with signaling pathways and genes involved in myocardial growth 29, and have adverse effects on ventricular mass through interactions between insulin, its receptor and the receptor of insulin-like growth factor 1 (IGF-1) in cardiac muscles 34. In addition, IR may decrease IGF-1 levels, which is negatively related to levels of growth hormone. Growth hormone may further contribute to an increase in LVM through its effect of proliferation and fluid retention on the heart 35. Moreover, IR may also be correlated to LVH through its inflammation-inducing property, as LVH has been associated with an increase in inflammatory markers 36.

Enlargement of the left atrium (LA) is considered to be the consequence of left ventricular (LV) diastolic dysfunction and subsequent LA volume or pressure overload 37. Previous studies have reported associations between LA enlargement and all-cause mortality 38 and future CV risk 39. In the present study, a high TyG index was associated with a large LAD. To the best of our knowledge, few studies have discussed the relationship between TyG index and LA size, while some studies have investigated the correlation between LA size and IR. In a study of Japanese hypertensive patients, Shigematsu *et al.* reported that IR determined by HOMA-IR was an independent predictor of echocardiographic LA enlargement 40. In addition, fasting blood glucose *per se* has also been associated with LA dimensions 41. Considering its relationship with LA size, LV diastolic function may help to explain the association between LA enlargement and IR. In a study of 1063 patients, Fontes-Carvalho *et al.* reported that HOMA-IR was independently associated with LV diastolic dysfunction, and that changes in diastolic function were present even before the onset of DM 42. In addition, Clarke *et al.* reported that pioglitazone, an IR-reducing medication, may improve myocardial insulin sensitivity and LV diastolic function 43. Triglycerides, another component of the TyG index, have also been correlated with LV diastolic dysfunction 44. In addition to the impact on diastolic dysfunction, inflammation and oxidative stress may also be the mechanisms underlying the association between IR and increase LA size 45,46.

In the present study, we found that a high TyG index was associated with a decline in LVEF. Subclinical LV systolic dysfunction has been reported to present in patients with metabolic syndrome but no history of heart failure, myocardial infarction and LVEF< 50% 47. In addition, in a large cohort study of 4425 individuals from the Cardiovascular Health Study, Banerjee *et al.* reported that fasting insulin levels were positively associated with the risk of heart failure 48. Moreover, in another longitudinal study, Cauwenberghs *et al.* found that hyperinsulinemia was related to a decrease in LVEF after follow-up for 4.7 years 32. There are some possible explanations for the association between IR and LVEF. Activation of the renin-angiotensin system (RAAS) and increased production of angiotensin II have been associated with derangement of glucose metabolism 49. In addition, interactions between IR and the RAAS may further contribute to cardiac dysfunction through different signaling transduction cascades 49 and by increasing the activity of angiotensin II in extracellular matrix deposition and cell proliferation 50. Moreover, IR and excess fatty acid have been associated with the deposition of intramyocardial lipids, and this has been suggested to result in subsequent myocardial fibrosis and systolic dysfunction 51.

Another important finding of this study is that the TyG index was negatively associated with ABI, a clinical parameter of atherosclerosis. Previous studies have demonstrated relationships between TyG index, IR and atherosclerosis. In a 1-year follow-up study of 366 patients, An *et al.* reported that IR was an independent predictor of atherosclerotic plaque progression in patients with coronary heart disease 52. In addition, Irace *et al.* reported that the TyG index was associated with carotid atherosclerosis even after adjusting for traditional risk factors of CV diseases 53. Several potential mechanisms may explain the associations between the TyG index, IR and atherosclerosis. Systemic factors in IR, such as hypertension, dyslipidemia and a pro-inflammatory state, may promote atherogenesis and plaque progression 54. At the cellular level, impaired insulin signaling may interfere with the normal function of vascular intimal cells that are involved in atherosclerosis, including macrophages, endothelial cells and vascular smooth muscle cells 54. In a study of 3587 Korean adults, Lee *et al.* reported that the TyG index was associated with increased arterial stiffness as assessed by baPWV 7. In addition, Won *et al.* also reported a positive association between the TyG index and arterial stiffness in a study of relatively healthy Korean subjects 55. In another recent study of 2830 elderly Chinese, Zhao *et al*. reported that the TyG index was associated with arterial stiffness as measured by cfPWV and baPWV, but that this was not associated with lower extremity atherosclerosis, as measured by ABI, after adjusting for CV risk factors 16. However, no significant association was found between the TyG index and baPWV in the present study. In a systematic review of pulse wave velocity, only age and blood pressure were identified as being important risk factors, while other CV risk factors including DM and triglycerides only showed an insignificant contribution to cfPWV 56. This may be because the TyG index is not correlated to arterial stiffness.

To the best of our knowledge, few studies have discussed the relationships between the TyG index and echocardiographic parameters. The strength of the present study is that we explored associations between the TyG index and cardiac remodeling and dysfunction as assessed using echocardiographic parameters, including LAD and LVEF. There are also several limitations to this study. First, as all of the participants were of Chinese ethnicity, our findings may not be generalizable to other ethnicities. Second, the HOMA-IR index was not included in the analysis, however the HOMA-IR index is the gold standard method to measure IR. However, the close relationship between HOMA-IR and the TyG index has been demonstrated in previous studies 5. In addition, the TyG index is more cost effective and easier to calculate in routine clinical practice. Third, the study design meant that we could not draw definitive conclusions with regards to long-term clinical outcomes and causal relationships. However, we believe that our findings highlight the importance of the TyG index in patients at risk of LV dysfunction and atherosclerosis. Further prospective studies are warranted to investigate these associations.

In conclusion, in this study, we demonstrated a significant association between a high TyG index and high LAD, low LVEF and low ABI. However, the TyG index was not closely associated with baPWV. These results suggest that the TyG index, as a simple indicator of IR, may reflect cardiac remodeling and dysfunction and atherosclerosis.

## Figures and Tables

**Table 1:** Comparison of baseline characteristics according to TyG index quartiles

Characteristics	Quartile 1 < 8.4 (n = 207)	Quartile 2 ≥ 8.4, < 8.8 (n = 207)	Quartile 3 ≥ 8.8, < 9.3 (n = 206)	Quartile 4 ≥ 9.3 (n = 203)
TyG index	8.1 ± 0.2	8.6 ± 0.1	9.0 ± 0.1	9.8 ± 0.5
Age (years)	63.3 ± 13.5	62.7 ± 12.4	60.6 ± 13.5	58.5 ± 13.0^ab^
Male (%)	54.6	57.5	59.7	63.1
Diabetes mellitus (%)	16.9	20.8	26.7	56.2^abc^
Hypertension (%)	67.1	75.8	76.1	77.3
Coronary artery disease (%)	15.2	18.0	22.9	24.1
Systolic blood pressure (mmHg)	133.2 ± 21.3	135.7 ± 19.5	137.8 ± 18.4	134.0 ± 18.1
Diastolic blood pressure (mmHg)	75.6 ± 11.9	76.9 ± 10.9	79.0 ± 11.1	77.6 ± 11.6
Mean arterial pressure (mmHg)	94.8 ± 14.0	96.5 ± 13.0	98.6 ± 12.5	96.4 ± 12.8
Body mass index (kg/m^2^)	24.6 ± 3.3	25.9 ± 3.8^a^	26.6 ± 3.6^a^	27.5 ± 3.9^ab^
ABI	1.12 ± 0.13	1.13 ± 0.11	1.12 ± 0.10	1.10 ± 0.13
baPWV (cm/s)	1717.7 ± 482.5	1746.8 ± 401.9	1804.2 ± 424.5	1739.2 ± 382.0
Laboratory parameters				
Total cholesterol (mg/dL)	180.4 ± 34.9	188.9 ± 41.5	196.0 ± 40.6^a^	204.5 ± 51.7^ab^
Hematocrit (%)	40.4 ± 4.8	40.4 ± 6.1	41.2 ± 5.0	41.1 ± 5.7
eGFR (mL/min/1.73 m^2^)	58.7 ± 16.8	58.3 ± 17.3	58.2 ± 20.7	57.2 ± 22.8
Echocardiographic data				
Observed/predicted LVM (%)	143.2 ± 38.2	147.5 ± 47.5	155.4 ± 46.8^a^	155.5 ± 48.0^a^
LA diameter (mm)	36.3 ± 6.3	37.9 ± 5.8	38.4 ± 6.1^a^	38.3 ± 6.0^a^
LVEF (%)	64.8 ± 11.9	64.6 ± 12.9	63.1 ± 13.0	61.8 ± 14.6

Abbreviations. TyG index, Triglyceride-glucose index; ABI, Ankle-brachial index; baPWV, brachial-ankle pulse wave velocity; eGFR, estimated glomerular filtration rate; LVM, left ventricular mass; LA, left atrial; LVEF, left ventricular ejection fraction. ^a^*p* < 0.05 compared with Quartile 1 ^b^*p* < 0.05 compared with Quartile 2 ^c^*p* < 0.05 compared with Quartile 3

**Table 2:** Determinants for echocardiographic parameters using multivariable stepwise linear regression analysis

Parameters	Multivariable (stepwise)	
	Unstandardized coefficient β (95% CI)	*p* value
Observed/predicted LVM (per 1%)		
TyG index (per 1)	4.401 (-0.540, 9.342)	0.081
Age (per 1 year)	-0.327 ( -0.584, -0.069)	0.013
Diabetes mellitus	8.957 (1.349, 16.566)	0.021
Mean arterial pressure	-0.573 (-0.819, -0.326)	< 0.001
eGFR (per 1 mL/min/1.73 m^2^)	-0.371 (-0.543, -0.199)	< 0.001
LA diameter (per 1 mm)		
TyG index (per 1)	0.926 (0.299, 1.533)	0.004
Sex (male *vs.* female)	1.931 (1.039, 2.822)	< 0.001
eGFR (per 1 mL/min/1.73 m^2^)	-0.053 (-0.075, -0.030)	< 0.001
LVEF (per 1%)		
TyG index (per 1)	-2.012 (-3.338, -0.686)	0.003
Age (per 1 year)	0.083 (0.010, 0.157)	0.026
Sex ((male *vs.* female)	-2.729 (-4.622, -0.835)	0.005
Hypertension	4.931 (2.807, 7.056)	< 0.001
Coronary artery disease	-5.426 (-7.789, -3.064)	< 0.001
eGFR (per 1 mL/min/1.73 m^2^)	0.104 (0.055, -0.153)	< 0.001

Values expressed as unstandardized coefficient β and 95% confidence interval (CI). Abbreviations are the same as in Table 1. Covariates in the multivariable model included TyG index, age, gender, diabetes mellitus, hypertension, coronary artery disease, mean arterial pressure, total cholesterol, hematocrit and eGFR.

**Table 3:** Determinants for abnormal ABI, baPWV using multivariable stepwise linear regression analysis

Parameters	Multivariable (stepwise)	
	Unstandardized coefficient β (95% CI)	*p* value
ABI (per 0.1)		
TyG index (per 1)	-0.147 (-0.279, -0.014)	0.030
Age (per 1 year)	-0.014 (-0.021, -0.007)	< 0.001
Sex (male *vs.* female)	-0.215 (-0.398, -0.031)	0.022
Coronary artery disease	-0.291 (-0.518, -0.063)	0.012
Total cholesterol (per 1 mg/dL)	-0.002 (-0.004, 0)	0.038
baPWV (per 100 cm/s)		
Age (per 1 year)	0.164 (0.147, 0.181)	< 0.001
Diabetes mellitus	1.322 (0.821, 1.822)	< 0.001
Mean arterial pressure (per 1 mmHg)	0.162 (0.144, 0.179)	< 0.001

Values expressed as unstandardized coefficient β and 95% confidence interval (CI). Abbreviations are the same as in Table 1. Covariates in the multivariable model included TyG index, age, gender, diabetes mellitus, hypertension, coronary artery disease, mean arterial pressure, total cholesterol, hematocrit and eGFR.
